# An intervention to reduce stigma and improve management of depression, risk of suicide/self-harm and other significant emotional or medically unexplained complaints among adolescents living in urban slums: protocol for the ARTEMIS project

**DOI:** 10.1186/s13063-022-06539-8

**Published:** 2022-07-29

**Authors:** Sandhya Kanaka Yatirajula, Sudha Kallakuri, Srilatha Paslawar, Ankita Mukherjee, Amritendu Bhattacharya, Susmita Chatterjee, Rajesh Sagar, Ashok Kumar, Heidi Lempp, Usha Raman, Renu Singh, Beverley Essue, Laurent Billot, David Peiris, Robyn Norton, Graham Thornicroft, Pallab K. Maulik

**Affiliations:** 1 The George Institute for Global Health, New Delhi, India; 2 All India Institute of Medical Sciences, New Delhi, India; 3 Dr.A.V. Baliga Memorial Trust, New Delhi, India; 4 Department of Inflammation Biology, Centre for Rheumatic Diseases, Faculty of Life Sciences & Medicine, King’s College London, London, UK; 5 University of Hyderabad, Hyderabad, India; 6 Young Lives India, New Delhi, India; 7 Institute of Health Policy, Management and Evaluation, Dalla Lana School of Public Health, University of Toronto, Toronto, Canada; 8 The George Institute for Global Health, Sydney, Australia; 9 University of New South Wales, Sydney, Australia; 10 Imperial College, London, UK; 11 Centre for Global Mental Health and Centre for Implementation Science, Institute of Psychiatry, Psychology and Neuroscience, King’s College London, London, UK

**Keywords:** Adolescent mental health, Slums, Other significant emotional or medically unexplained complaints, Depression and increased risk of self-harm/suicide, Primary healthcare worker, Anti-stigma campaign, Electronic decision support systems, India, Randomised control trial

## Abstract

**Background:**

There are around 250 million adolescents in India. Adolescents are vulnerable to common mental disorders with depression and self-harm accounting for a major share of the burden of death and disability in this age group. Around 20% of children and adolescents are diagnosed with/ or live with a disabling mental illness. A national survey has found that suicide is the third leading cause of death among adolescents in India. The authors hypothesise that an intervention involving an anti-stigma campaign co-created by adolescents themselves, and a mobile technology-based electronic decision support system will help reduce stigma, depression, and suicide risk and improve mental health for high-risk adolescents living in urban slums in India.

**Methods:**

The intervention will be implemented as a cluster randomised control trial in 30 slum clusters in each of the cities of Vijayawada and New Delhi in India. Adolescents aged 10 to 19 years will be screened for depression and suicide ideation using the Patient Health Questionnaire (PHQ-9). Two evaluation cohorts will be derived—a high-risk cohort with an elevated PHQ-9 score ≥ 10 and/or a positive response (score ≥ 2) to the suicide risk question on the PHQ-9, and a non-high-risk cohort comprising an equal number of adolescents not at elevated risk based on these scores.

**Discussion:**

The key elements that ARTEMIS will focus on are increasing awareness among adolescents and the slum community on these mental health conditions as well as strengthening the skills of existing primary healthcare workers and promoting task sharing. The findings from this study will provide evidence to governments about strategies with potential for addressing the gaps in providing care for adolescents living in urban slums and experiencing depression, other significant emotional or medically unexplained complaints or increased suicide risk/self-harm and should have relevance not only for India but also for other low- and middle-income countries.

**Trial status:**

Protocol version – V7, 20 Dec 2021

Recruitment start date: tentatively after 15th July 2022

Recruitment end date: tentatively 14th July 2023 (1 year after the trial start date)

**Trial registration:**

The trial has been registered in the Clinical Trial Registry India, which is included in the WHO list of Registries (https://www.who.int/clinical-trials-registry-platform/network/primary-registries) Reference No. CTRI/2022/02/040307. Registered on 18 February 2022.

The tentative start date of participant recruitment for the trial will begin after 15th July 2022.

**Supplementary Information:**

The online version contains supplementary material available at 10.1186/s13063-022-06539-8.

## Background

There are around 250 million adolescents in India [1]. The 2016 National Mental Health Survey in India estimated that the prevalence of any mental illness among adults is about 15%, with nearly 150 million people in need of treatment [2]. The survey also found that adolescents are vulnerable to stress, depression, and increased risk of self-harm/suicide, which are leading causes of death and disability for this group in India. The prevalence of depression, stress, and substance use disorders accounts for most of the mental illnesses and is estimated to be around 10% [3]. Previous research has shown that India has relatively high suicide rates [4] that are particularly high among adolescents and young adults, and with wide variation in suicide rates across the country [5]. Data have shown that around 20% of children and adolescents experience a disabling mental illness [6].

### Treatment gaps for mental disorders in India among adolescents

The treatment gap (i.e. the difference between the number of people with a mental disorder and the number receiving appropriate treatment) for adults as well as adolescents with common mental disorders is large—estimated to be 75–95% in India, and further gaps in the quality of care are even more marked with around 4% of people with major depressive disorders receiving guideline-recommended care [7–9]. The reasons for these gaps are multifactorial, and demand-side barriers include limited awareness among many communities, low identification of individuals with mental illness, stigma, discrimination and negative community perceptions about mental health and help-seeking [10, 11]. Supply-side barriers include a lack of trained mental health professionals and lack of availability of specialist services [12, 13]. The availability of psychiatric health care professionals in high-income countries is approximately 75 to 120 times greater than in India [14] with the density of health care staff being much lower in rural areas.

### Need for community-based mental health services for adolescents

The Adolescents’ Resilience and Treatment nEeds for Mental health in Indian Slums (ARTEMIS) study seeks to address adolescent mental health through a community-based health service programme to deliver technology-enabled mental health services for disadvantaged adolescents in urban slums. Adolescents in slums face many stressors including poverty, academic stress, difficulties in relationships [15], unemployment, lack of education of parents [16], housing structure/living conditions [17], interpersonal violence (among males and females), low access to mental health care [18], and significant school dropout rates which increases their vulnerability. They also lack access to adequate support facilities, including health services. The ARTEMIS study also attempts to understand and quantify the stigma towards mental illnesses among adolescents in urban slum communities and to evaluate the impact of strategies to reduce this stigma through an extensive anti-stigma campaign.

In terms of interventions, a meta-analysis of community-based programmes for low-income urban youth indicated that for depression interventions a combined person-centred and environmental approach (such as involving families when appropriate) had the best effect [19]. However, existing studies are of varied quality and no age, gender, or geographical area-specific intervention data are available [20]. A review of stigma research found that adolescents prefer educational materials in either electronic or paper form as the main sources of information related to stigma, but the studies are limited and are non-existent from India or other low-and middle-income countries (LMICs) [21]. Two Indian studies have shown that basic mental health care delivery is possible by training lay health workers. However, such interventions involve recruiting additional personnel, which may affect scalability and generalizability for low-resource PHC settings [22, 23]. Other research has also shown that training primary health workers in delivering basic mental health care for stress, depression, and increased risk of self-harm/suicide is possible when suitably supported by evidence-based tools such as an anti-stigma campaign and technology-enabled mental health services delivery strategy [24].

To our knowledge, this trial is the first to focus on adolescents with mental illness in urban slum communities in India. The ARTEMIS study aligns with the Indian Government’s *Rashtriya Kishor Swasthya Karyakram (RKSK, National Adolescent Health Programme)* that focuses on adolescent health [25], and the National Mental Health Programme that emphasises the need for the delivery of innovative strategies by strengthening existing mental health care systems [26], as well as with WHO’s Comprehensive Mental Health Action Plan 2013-30 [14] at the international level. The study also aligns with the UN Sustainable Development Goal (SDG) of Good Health and Wellbeing by providing inclusive healthcare for adolescents in particularly disadvantaged communities and is directly related to the SDG Target 3.4.2 to reduce suicide rates [27]. To support these national and international programmes and goals, it is important that affordable, accessible, and effective treatments are available to tackle mental disorders, to strengthen individual resilience [28], to provide mental healthcare support and to create avenues to discuss mental health problems in a non-stigmatising manner.

### Mobile health (mHealth) strategies to support primary healthcare

Mobile health (mHealth) technologies are used to address access and treatment gaps for health conditions in primary healthcare settings, especially mHealth applications that seek to strengthen the capacity of the workforce using electronic decision support systems (EDSS). With the increased penetration of mobile devices, especially smart phones in India [29], the integration of EDSS into mobile devices (mobile phones, smartphones and tablets), can increase the reach into Indian communities. Studies conducted in high-income country settings have demonstrated the benefits of an EDSS in managing various health conditions, including mental health, especially for tools that use evidence-based algorithms to provide individualised advice at the point of care [30]. However, very few studies have been able to demonstrate improvements in clinical outcomes and most trials of such tools have resulted in improved processes of care only [31, 32]. In India, a systematic review of mHealth interventions found low-quality evidence on the efficacy, acceptability, and cost-effectiveness of such initiatives [33]. The ARTEMIS study will be contributing to filling in this gap by employing rigorous scientific evaluation to test the effectiveness of the mhealth component by using appropriate methods.

### Developing the intervention

In order to address these research gaps, the study’s aim is to develop and evaluate a complex intervention incorporating an anti-stigma campaign and a mobile technology-based EDSS to facilitate the delivery of mental health care for depression, other significant emotional or medically unexplained complaints, and increased risk of self-harm/suicide among adolescents living in urban slums. ARTEMIS will adapt the SMART Mental Health intervention [34] for adolescents (10–19 years) living in urban slums, in Vijayawada and New Delhi, and conduct a cluster randomised control trial (cRCT) to determine whether this strategy will increase remission rates for adolescents with depression, or increased risk of self-harm/suicide.

The two core components of the ARTEMIS intervention will be:

1. An anti-stigma campaign to improve community behaviours toward adolescents with depression or increased risk of self-harm/suicide; and
2. Implementation of a mobile device-based decision support system to improve the treatment of adolescents at high risk of depression or self-harm/suicide leading to higher remission rates from depression and reduced suicide risk.

### Anti-stigma campaign materials

Strategies used in the anti-stigma campaign straddle the three pillars of stigma namely, problems of knowledge (ignorance), problems of attitudes (prejudice) and problems of behaviour (discrimination) [35]. This includes the creation of educational materials to target inaccurate knowledge and stereotypes; interpersonal contact with members of a stigmatised community; and public protests against those who stigmatise other groups [21]. Reviews of anti-stigma campaigns have shown that interpersonal contact and to a lesser degree, educational materials, have been variably effective, with most evidence coming from high-income countries [36].

An important element of the ARTEMIS project is to establish an Adolescent Expert Advisory Group (AEAG) to guide researchers on all aspects of the project design, development and implementation. Three AEAG groups each will be formed in both of the sites; one a mixed group of younger adolescents (10 to 14 years) and two sex-segregated groups of older adolescents (15 to 19 years). The dynamics of engaging with adolescents and retaining them in a programme, especially in a LMIC, will be tested in consultation with the AEAG, as part of related doctoral research. Use of tools such as photo voice, social media, to discuss mental health issues will also be explored as part of the related doctoral research.

Formative research will be conducted to inform the development of information, education and communication (IEC) materials drawing on earlier research and adapting to the local context [37]. Preferences and suggested strategies will be sought from the community during the formative phase which includes, the adolescents themselves, and adults including parents and non-physician health workers (NPHWs), to implement an anti-stigma campaign for adolescents that is feasible and acceptable. These strategies will be designed by the AEAG and the intervention team to address the lack of awareness, misinformation about mental health among the slum communities, and will use education-based, interpersonal, and social contact approaches to increase knowledge, and reduce stigma, discrimination and social isolation related to depression and suicide/self-harm. The content will be developed in Telugu and Hindi, the most commonly spoken languages in the two study regions.

The content of the anti-stigma campaign will include the following:

1. Printed IEC materials: brochures, flipbooks, and posters on signs and symptoms of depression, anxiety, substance use, and suicide risk/self-harm in adolescents; the need for seeking treatment and treatment/management options apart from medicines and psychotherapy; and stigma related to mental health prevalent in the community. Vignettes and infographics will be included in the brochures. Brochures and flipbooks will be used during door-to-door campaigns and community meetings during the intervention phase to raise mental health awareness and discuss problems related to stigma. NPHW such as Accredited Social Health Activists (ASHAs), Auxiliary Nurse Midwives (ANMs), and field staff will be involved in distributing these materials. The posters and pamphlets may also be shared with ward offices (a local authority area, typically used for electoral purposes), schools and urban primary health centres (UPHC) and displayed on their walls or notice boards.
2. Videos: adolescents who experienced stress, depression, and thoughts of suicide/self-harm will talk about their experiences using video narratives of their lived experiences. Research has found such social contact –both direct and indirect (in the form of videos) with people with lived experiences to be beneficial in reducing stigma in the general public [38, 39]. These lived experience videos will be screened in the slums and discussed with adolescents, parents and other stakeholders as part of the anti-stigma campaign. Informed consent from the adolescents/their parents/guardians and assent will be sought before making these videos.
3. Street theatre/plays: another method of indirect social contact that ARTEMIS will employ are street plays performed by adolescents themselves, supported by local street theatre groups highlighting mental health issues and help-seeking, as research shows that theatre are powerful mediums [40].
4. Use of multimedia: two short animations on stigma concepts in line with that proposed by AEAG members will be produced and screened.

### mHealth-based EDSS

Separate EDSS modules will be developed for NPHW and UPHC doctors. The software will be developed on an Android platform, optimised for 7-inch tablets. The EDSS will include a screening tool based on the Patient Health Questionnaire (PHQ-9) [41, 42] which has been validated in India [43]. The PHQ-9 is available in the two vernacular languages spoken in Vijayawada and New Delhi and has been validated as an excellent tool for screening depression among adolescents [44]. The diagnostic and management guidelines that will be used by the UPHC doctor are based on WHO’s Mental Health Gap Action Programme- Intervention Guide (mhGAP-IG) [45] and focus on three conditions: depression, other significant emotional or medically unexplained complaints and self-harm/suicide. The mhGAP-IG is in English. The algorithm will be modified based on multiple iterations and feedback from the research team and a psychiatrist and has been validated in a previous mixed methods study [46]. The treatment algorithm provides guidelines about both pharmacological and psychological treatment including referral to a mental health specialist. A traffic-light dashboard called the priority listing app will also be included to support NPHWs to follow up high risk individuals and act on the treatment provided by the doctors.

The implementation of the mobile technology-based EDSS will include the following:

1. Trained interviewers will administer the PHQ-9 to adolescents aged 10 to 19 years in the selected slum clusters to screen for depression and suicide/self-harm risk. They will use a tablet-based questionnaire to generate composite scores to identify ‘high-risk’ adolescents.
2. NPHWs and doctors will be trained by the research team before the beginning of the intervention. Training of NPHWs and UHC doctors will be provided by in-person training and via the virtual conferencing platforms developed earlie r[24]. The NPHWs will then provide basic supportive advice such as engaging oneself in hobbies, strengthening social contact, and so on, and a referral to the UPHC doctor. High-risk adolescents will be electronically referred via the mHealth platform and provided with a paper referral card to take to the doctor.
3. The UPHC doctor will then review adolescents at high risk as part of a health camp organised in the slum or examine the patient who may directly visit the UPHC. Data from adolescents at high risk will be uploaded, with their consent, to a secure health record using the Open Medical Record System (MRS) open source, electronic health record system.
4. A decision support application based on WHO’s mhGAP-IG algorithm will be integrated in a tablet device and that will be used by UPHC doctors to diagnose and manage patients experiencing depression, other significant emotional or medically unexplained complaints and or having self-harm/suicide ideation. Doctors will use the depression, other significant emotional or medically unexplained complaints and self-harm/suicide modules of mhGAP-IG, especially related to adolescents. Adolescents at high risk of requiring medication can access these directly from the UPHC or purchase low-cost generic medication from a private pharmacy.
5. For adolescents with complex needs, consultation with a specialist psychiatrist will be available as needed. The psychiatrist will also conduct case reviews via phone and/or video conferencing with the UPHC doctors to enhance their management skills. Alternatively, such adolescents can be referred by the UPHC doctor to a psychiatrist in the next tier of the public health system.
6. NPHWs will conduct follow-up visits and assess treatment adherence using the traffic light system to guide them about the status of each high-risk adolescent catered to by a particular NPHW.

### Potential contributions to the field

- This study will contribute to the literature on adolescents living in urban slums diagnosed with/ or live with mental disorders in low- and middle-income settings and will assess the potential impact of implementing an anti-stigma campaign about mental illness that has been co-created with adolescents
- This study will elucidate the barriers and facilitators for help-seeking by adolescents living in slums and diagnosed with/living with depression, other significant emotional or medically unexplained complaints or suicide/self-harm
- This study will offer an understanding of the effect of using a combination of task sharing, an anti-stigma campaign and a technology-enabled mental health service delivery model to tackle depression, other significant emotional or medically unexplained complaints or increased suicide risk/self-harm among adolescents living in urban slums.
- The anti-stigma campaign will be one of the largest of such programmes for adolescents living in urban slums conducted in a LMIC.

## Research methods

### Aim

The study aims to test clinical effectiveness and cost-effectiveness of the implementation strategies to identify and reduce depression and suicide risk among adolescents living in urban slums. The study hypothesises that**:**

1. A community-based anti-stigma campaign will lead to significant improvements in community behaviours toward adolescents with depression, other significant emotional or medically unexplained complaints and increased risk of self-harm/suicide; and
2. A mobile device-based decision support system will improve the treatment of adolescents at high risk of depression, other significant emotional or medically unexplained complaints and increased risk of self-harm/suicide and lead to higher remission rates from depression and reduced suicide risk.

### Study design

**Fig. 1:**
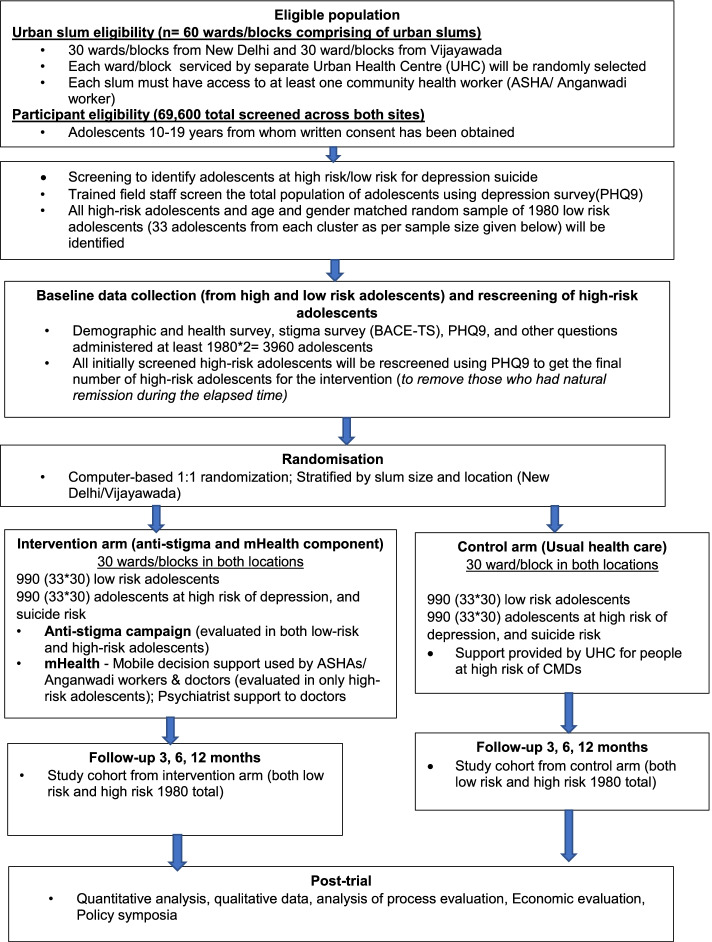
Study schema

A cluster randomised, control trial (cRCT) involving UPHCs in urban slum clusters in the cities of Vijayawada (in the state of Andhra Pradesh) and New Delhi will be conducted. The adolescent population will be screened to identify two cohorts: (1) adolescents at ‘high-risk’ of depression, suicide/self-harm; and (2) a random sample of adolescents not at high-risk. These will comprise the evaluation cohorts and they will be followed up for 12 months. Detailed process and economic evaluations will also be conducted (refer to Fig. 1 which outlines the study schema).

### Setting

The study will be conducted in two locations: slums clusters of Vijayawada and New Delhi. New Delhi is in the north of India, is the capital of the country and is the largest metropolitan city (population about 20 million) in India. Vijayawada is the second largest city in the south Indian state of Andhra Pradesh (population about 2 million). At each site, 30 slum clusters will be selected. For this study, a slum cluster is defined as slums within wards or geographical areas identified as slums/resettlement colonies. A ward is a local authority area, typically used for electoral purposes. In certain cities of India, such as Mumbai and New Delhi, a ward is an administrative unit of the city region; a city area is divided into zones, which in turn contain numerous wards. The slum clusters (constituting 1–5 slums) in Vijayawada, Andhra Pradesh and in New Delhi, under the Municipal Corporation of Delhi, will be selected from slums located within a radius of 60 km from the field office in both cities based on discussions with study collaborators and team members with prior experience working in these areas and will be selected to avoid contiguity. Each slum cluster will have a population of approximately 8000 and will be serviced by at least one UPHC. In Vijayawada, a few of the clusters selected lie in peri-urban areas but are informally considered as part Vijayawada both by local civil society organisations as well as by the Vijayawada municipality and are serviced by UPHCs and by the municipality.

### Eligible population

#### High-risk cohort

All consenting adolescents aged 10 to 19 years will be eligible for screening for depression, or suicide/self-harm risk. The screening will help to identify both high-risk and non-high-risk cohorts. High risk is defined as presence of at least one of the two conditions:

1. High risk of depression based on PHQ-9 score ≥10.
2. Positive response (score ≥2) to the suicide risk question on the PHQ-9.

Field investigators will continue screening until they have identified about 990 adolescents per location at high risk of depression or self-harm/suicide. This will form the ‘high-risk’ cohort. Because there will be some delay (up to 12 weeks) between screening and randomisation the high-risk adolescents will be rescreened to assess if they still meet the inclusion criteria prior to randomisation as literature suggests that around 25% of individuals initially identified at high risk will no longer meet the high-risk criteria at three months [47]. Only those adolescents who continue to remain at high risk during re-screening will form part of the high-risk cohort.

#### Low-risk general adolescent cohort

A second cohort of adolescents per slum cluster not at high risk for depression and/or self-harm/suicide will be identified by selecting a random sample from the remaining screened population matched by gender and age per slum. This will form the ‘low-risk’ cohort. The numbers per slum will be in proportion to the high risk per slum by gender and age.

### Exclusion criteria

The following adolescents will not be included in the study.

1. Adolescents with severe physical or mental ill-health that would prevent them from giving consent or participating in the study
2. Adolescents or their guardians who do not provide informed written consent
3. Adolescents who are temporary residents of the slums such as visiting relatives/friends

The implementation team will comprise of researchers, project staff, and field investigators. Project staff will continuously monitor the trial implementation and support field staff, NPHW and UPHC doctors. Research staff will continuously monitor the data and provide feedback to the field staff about any deviations. In addition, 10% of data will be re-assessed by the designated field staff to ensure quality control, and corrective steps will be taken as needed. They will ensure maximum inclusivity of adolescents who manifest either physical or mental illness and field investigators will be provided training in lines with the adapted Guidelines on Disability Inclusion in Research [48] to ensure that as many adolescents as possible are included in the study.

### Randomisation

Randomisation will be conducted at the level of the slum cluster, in a 1:1 ratio to intervention or control using a central allocation sequence. Natural remission variation between screening and rescreening may be used if deemed appropriate for stratification prior to randomisation. The clusters will be stratified by location, average remission rate for depression between screened and re-screened population per slum cluster and median population of each slum cluster. The slum cluster selected under each intervention and control arms will be non-contiguous to avoid contamination. The random allocation will be performed using a software-based random number generator by a statistician not involved in study conduct or analysis. To maintain blinding, the unblinded statistician will generate the randomisation list and share it directly with field staff. The blinded study statistician as well as the principal investigator will remain blinded until data lock.

### Intervention components

**Fig. 2:**
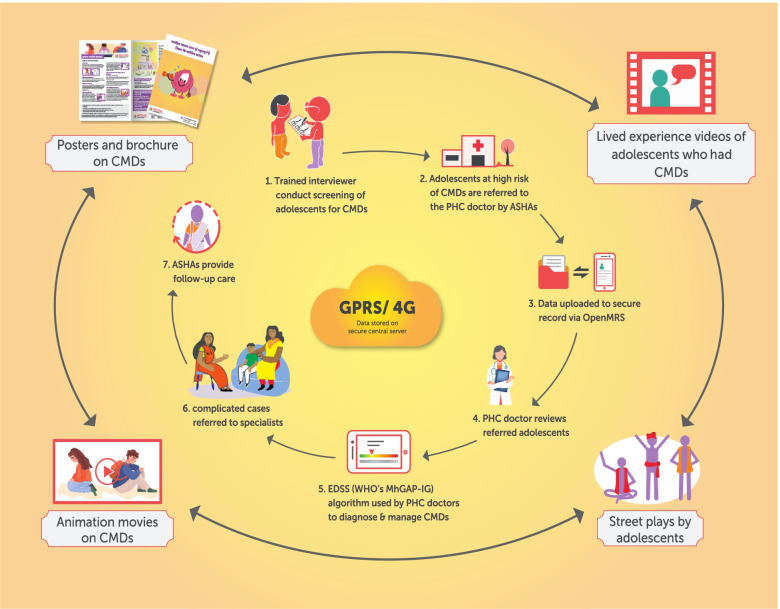
Intervention prototype of ARTEMIS study

The two core intervention components of the ARTEMIS described above include the community anti-stigma campaign and electronic decision support system. Figure 2 outlines how the different components of the trial work together.

The anti-stigma campaign will be implemented immediately post-randomisation and will continue throughout the intervention period, which will include different strategies of creating awareness using multimedia approaches, having a social contact approach (where people with lived experiences share their experiences through videos, in-person talks) and delivering these strategies.

### Control arm

The control arm slum cluster will receive usual care (currently the usual care practice involves very little support for mental disorders and only those with severe disorders are referred to a mental health professional) from their UPHC and general psychoeducation through brochures and pamphlets only. As part of good clinical practice, adolescents identified at high risk for depression or self-harm/suicide will be advised to consult a doctor or mental health professional for further diagnosis and treatment. If any adolescent is identified with severe depression, (a score of >=15 on PHQ9) and/or at high risk of suicide (suicide score >=2) during the screening, they and their parents/guardians will be advised to seek professional help immediately. A list of health facilities (with contact details) where such treatment is available will be provided to them. Further, the implementing team will request NPHW to follow up with the adolescent and their families more intensively and recommend them to visit a doctor/psychiatrist immediately. The implementation team will check to see whether the NPHW has made follow-up visits to such adolescents.

### Both arms

For all UPHCs, that fall within the study sites, the implementing team will liaise with government and private pharmacies to ensure the availability of appropriate medications. We will also explore collaborative strategies with the Ministry of Health and Family Welfare of both states (Andhra Pradesh and Delhi), to enhance the availability of common psychotropic medications for adolescents as per the Essential Drugs List in the UPHCs. Detailed contact information will be collected from all participants, such that follow-up can be done. The study participants will be constantly followed up for their wellbeing by the NPHWs and will also provide a great deal of motivation and encouragement to them to seek help for their mental illness on a regular basis.

### Data collection

Independent field investigators, blinded to intervention allocation, will be involved in data collection at each phase of the study. Quantitative data on stigma perceptions related to mental health, depression will be collected prior to randomisation (Time 0) and then subsequently at 3, 6, 12 months of the intervention. All data including clinical data of study participants will be captured on tablets, de-identified and saved in a secure server.

- *Time - 0 month (pre-randomisation):* The names of those adolescents at high-risk will be shared with trained interviewers who will then administer a detailed questionnaire that will enquire about socio-demographic characteristics, treatment history, history of any mental illness, family history of any mental illness, social support from friends and families, stressful events experienced in the last one year, resilience, history of any co-morbid major physical illnesses.. Questions related to costs incurred on treatment will be directed to the parents/guardians of the adolescents.
- *Time – 3, 6, and 12 months (intervention phase):* Data will be collected from the study cohort from the intervention arm and the study cohort from the control arm (both low risk and high risk) across all the slum clusters by trained interviewers. Questionnaires administered will be the Patient Health Questionnaire (PHQ9), Knowledge, Attitude, and Behaviour (KAB) scale [49] and Barriers to Access to Care Evaluation- Treatment Stigma (BACE-TS) [50], Connor-Davidson Resilience Scale (CD-RISC - 10) [51] at these time points.

### Tools used for data collection

i) PHQ9—The PHQ9 consists of 9-items. The final score is calculated by assigning scores of 0, 1, 2, and 3, to the response categories of “not at all”, “several days”, “more than half the days”, and “nearly every day”, respectively. PHQ-9 total score for the nine items ranges from 0 to 27, and scores 5–9 indicate mild, 10–14 indicate moderate, 15-19 indicate moderately severe, and 20–27 indicate severe depression. This instrument will provide a reliable estimate of depression in the communities and also the proportion among the screen positive individuals who have accessed mental health services. A cut-off score of ≥5 is indicative of mild depression needing further follow-up and assessment by the NPHWs, whereas a score of ≥10 needs clinical assessment by a PHC doctor.
ii) CD-RISC – 10 scale—This scale consists of 10 items measuring resilience. Responses are rated on a 5-point Likert scale, ranging from 0 (not true at all) to 4 (true nearly all the time). Each score has a minimum score of 0 and a maximum score of 4. The total scores are calculated by adding the scores of all 10 items. Higher scores suggest greater resilience and lower scores suggest less resilience, or more difficulty in bouncing back from adversity.
iii) KAB scale—KAB is used to measure the knowledge, attitude and behaviour related to mental health, and is a 16-item questionnaire. 12 of the items ascertain mental health knowledge, attitude and behaviours to understanding stigma. It has two subgroups—the behaviour subgroup and the knowledge and attitude subgroup. The behaviour subgroup is based on the Reported and Intended Behaviour Scale which uses a 5-point Likert scale (ranging from ‘agree strongly’ which scored 1, to ‘disagree strongly’ which scored 5). Higher scores suggest increased stigma, except for those questions which had a negative connotation. The knowledge and attitude subgroups were identified based on discussion with experts hence does not have the properties of a scale
iv) BACE – TS scale—This is a 12-item questionnaire with a four-point Likert scale, with scores ranging from 0 to 4, which asks questions on the stigma associated with seeking help for mental illnesses. Higher scores suggest higher stigma.

**Fig. 3:**
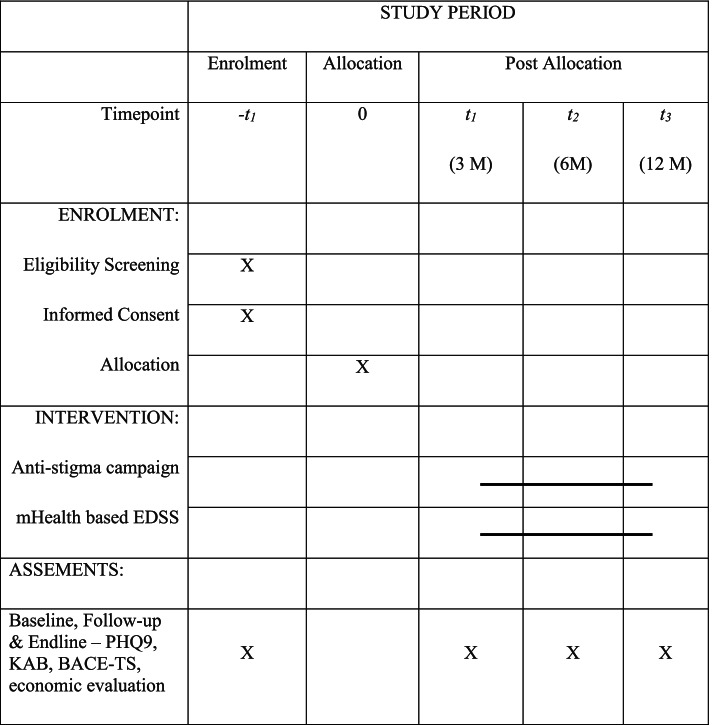
Study participants’ timeline of enrolment, eligibility screening, allocation, intervention and assessments at four timepoints

The SPIRIT 2013 statement, 33-item checklist and figure [52] is being used to schematically represent the study participants’ timeline of enrolment, eligibility screening, allocation, intervention and assessments at four timepoints (please see Fig. 3) and to guide the overall standards of a cRCT.

#### Anti-stigma component (combined high- and low-risk cohorts)

##### Primary outcome

1. Change in mean behaviour scores of adolescents at 12 months using the KAB scale

##### Secondary outcomes

1. Change in mean knowledge and attitude scores and change in stigma perceptions of adolescents as assessed by Barriers to Access to Care Evaluation- Treatment Stigma (BACE – TS) Subscale. Change in attitude of parents demonstrated through qualitative interviews of parents selected through purposive sampling will also be captured. The qualitative tool development, data collection and analysis will be done by The George Institute’s qualitative researcher.
2. Change from baseline in mean stigma scores of adolescents

#### mHealth component (high-risk cohort)

##### Primary outcome

1. Proportion achieving remission (defined as PHQ-9 <5, and/or suicide risk score <2) at 12 months.

##### Secondary outcomes

1. Mean difference in PHQ-9 scores of adolescents at 12 months.
2. Proportion who have visited a doctor at least once between randomisation and 12 months

### Statistical considerations

For high-risk adolescents, assuming a 50% remission rate in the control arm at 12 months based on published data [53], and 30 slum clusters per intervention location, at least 27 subjects per slum cluster are needed to detect a 15% absolute improvement in the intervention arm (65% intervention vs 50% control) at 12 months with 90% power. This assumes a 2-sided significance level of 0.05 and an intra-class correlation coefficient (ICC) of 0.1 based on pilot data [25]. To account for up to 20% of participants lost to follow-up, we will aim to enrol at least 33 participants per slum cluster for a total sample size of 1980 adolescents (60 clusters × 33 adolescents). Assuming a prevalence of 3% of adolescents with depression or increased risk of self-harm/suicide (48), we will therefore aim to screen about 1100 adolescents in each slum cluster. Further assuming that 5% of eligible adolescents will refuse to participate, we aim to screen 1160 adolescents in each slum cluster i.e. a total of 69,600 adolescents (60 slum clusters × 1160 adolescents).

Primary analyses will be conducted at the participant level using either random-effect generalised linear mixed models or generalised estimating equations adjusted for clustering. For the primary outcome, remission at 12 months differences will be assessed using a log-binomial (log link function) or logistic regression (log link function) including a random cluster effect. If using generalised estimating equations, the effect of clustering will be accounted for using a repeated cluster effect with a compound-symmetry variance-covariance structure. The intervention effect will be estimated as odds ratio and corresponding 95% confidence interval.

Sub-group analyses will be conducted according to UPHC-level characteristics (size, location and health service characteristics) and patient-level characteristics (demographic factors and clinical factors e.g. depression severity at baseline). The primary outcome will be assessed at 12 months (and reported as secondary outcomes at 3 and 6 months).

Continuous outcomes such as knowledge and behaviour scores of adolescents will be analysed in a similar manner but using generalised linear mixed model with identity link function in place of logit or log link function and with the baseline value of the score included as a covariate in the model. A pre-specified analysis plan including sensitivity analyses, potential covariate adjustments, analyses for secondary endpoints and detailed assumptions (e.g. missing data handling) will be developed prior to unblinding. To ensure that the analysis is not influenced by potential results, all analyses will initially be programmed using randomly scrambled groups. The real group allocation will be used once the statistical analysis plan is finalised, the database locked, and all analyses programmed.

### Economic evaluation

A trial-based and a modelled evaluation of long-term costs and outcomes will be conducted from a health system perspective. Intervention costs will include salaries, training, and equipment. A within-trial comparison of costs (e.g. visits, medications) will enable estimation of potential cost-offsets associated with the intervention. Average differences in utility observed between treatment arms will be determined using the Short Form 6-Dimension (SF6D), which will be calculated using the Brazier algorithm [54] from the Short Form 36 (SF36) questionnaire. The SF6D is a utility instrument that is derived from the SF36 which is a standard tool for measuring health-related quality of life in economic evaluations [55]. It includes a specific domain for assessing mental well-being, has routinely been used in adolescent mental health research and is validated for use in India [55]. To capture costs and outcomes beyond the trial, a decision-analytic model will be developed to enable long-term morbidity and quality of life to be simulated. Incremental costs per Quality Adjusted Life Years gained will be determined. Sensitivity analyses will determine the robustness of the assumptions of the model.

### Process evaluation

Processes will be monitored continuously throughout the intervention phase. A formal evaluation will be carried out at the end of the intervention using Michie’s Behaviour Change Theory [56] as the over-arching framework to guide our understanding of the intervention implementation and impact. This theory assesses the capability, opportunity, and motivation of adolescents, PHC workers and doctors to engage with the intervention. The researchers will take a case study approach in which a purposive sample of clusters will be selected. In addition to Behaviour Change Theory, the MRC framework for evaluating complex interventions [57] and the RE-AIM (Reach, Effectiveness, Adoption, Implementation, and Maintenance) framework [58] will be used to evaluate the implementation. Qualitative data about the experiences of the adolescents, community participants, UPHC doctors, NPHW, and other key stakeholders will be gathered by researchers through focus groups and in-depth interviews until thematic saturation is reached. For participants in the mHealth intervention arm, the mHealth system will capture health services usage analytic data to assess intervention fidelity. A mixed methods analysis will be conducted informed by the Medical Research Council UK (MRC) guidelines [57]. The process evaluation will assist in the interpretation of the ARTEMIS study results, to understand barriers and facilitators in implementation of the intervention components, explain what aspects work or need modification in future trials, and evaluate whether the intervention was implemented appropriately, and as planned.

### Trial status

Protocol version – V7, 20 Dec 2021

Protocol submitted to: Clinical Trial Registry of India (CTRI)

Trial status: Study approved by CTRI (Reference Number CTRI/2022/02/040307, http://ctri.nic.in/Clinicaltrials/pmaindet2.php?trialid=47111&EncHid=&userName=ARTEMIS-%20CTRI/2022/02/040307).

Trial registration date: 18 February 2022

Recruitment start date: tentatively after 15th July 2022

Recruitment end date: tentatively before 14th July 2023 (one year following recruitment start date)

### Ethical considerations

The study is approved by the George Institute for Global Health India. Approval of the ARTEMIS project from the Health Ministry’s Screening Committee (HMSC), Indian Council for Medical Research (ICMR) has also been received (ID 2020-9770). The study also received ethical clearance from Research Governance and Integrity Team, Imperial College, London on 8^th^ June 2022 (ICREC Reference number: 22IC7718). Any protocol amendment will be shared with the ethics committee approval. If there are protocol deviations, those will be reported to the trial registry too. Any protocol amendment following the start of the trial will also be reported to the trial registry.

Preliminary discussions about the study and interventions with each ward member, PHC doctors and NPHWs were conducted during the initial slum cluster mapping and household listing exercises. Informed written consent will be obtained from all participants who are aged 18 and 19 years and from parents/guardians of under-aged adolescents (10 to 17 years) along with assent from these underage adolescents, by trained interviewers at the time of screening for high-risk populations and prior to administering the detailed questionnaire at baseline and for focused group discussions and interviews. Any individual identified at imminent risk of suicide at baseline will be advised to seek professional help from a doctor/psychiatrist immediately whose contact details would be provided. All data collection and reporting will be compliant with ICMR guidelines [59]. Independent study monitoring will occur for a subset of participants. Data will be de-identified, stored on George Institute India servers, and held in strict compliance with Good Clinical Practice guidelines using our standard operating procedures for data security, confidentiality, backup, and audit trails. As required, all raw data and any derived datasets will be stored at the George Institute India Hyderabad office for at least 10 years from study completion.

### Adverse event reporting

Serious adverse events will be recorded after the start of the intervention phase. A serious adverse event is defined as death due to any cause in the intervention or control arm, hospitalisation due to psychiatric disorders, or a history of self-harm or attempted suicide during the intervention period. These events will be captured using a standardised case report form and reported to an independent Data Safety Monitoring Committee Chairperson on a monthly basis and Ethics Committee Chairperson within 24 h of the implementing team being aware of the incident. This data will be analysed during the Data Safety Monitoring Committee meetings that will be held on a quarterly basis.

### Stopping rule

In order to address safety concerns, at least one interim analysis will be conducted with results reviewed by the Data Safety and Monitoring Committee (DSMC). If the interim analysis shows a benefit from the intervention the study will proceed as planned as the sustained effect at end of the study will remain an ongoing question. A recommendation to discontinue the study prematurely will be based upon there being clear evidence that the intervention provides harm. Although no formal/ binding stopping rules are suggested, an increase in the proportion of participants with a suicide risk score >2 at 3 and 6 months in the intervention arm of 3 standard deviations (a 2-sided *p* value < 0.0027) or more would be regarded as strong evidence of early harm. The DSMC will reveal the unblinded results to the Trial Steering Committee if, taking into account both statistical and clinical issues and exercising their best clinical and statistical judgement, the unblinded results provide sufficient evidence that the trial treatment is on balance harmful.

### Programme milestones

Ethical approval, site approvals and modification of IEC materials will be completed 15 months after the commencement of the study. Screening of eligible adolescent population and baseline interviews of study participants will be completed by 20 months. Randomisation of PHCs into the intervention and control arm will be done in May 2022. Implementing the intervention (anti-stigma campaign and EDSS decision making), follow-up and endline data collection will be completed by 34 months. Publications will be done to highlight various aspects of the project throughout the project period and especially to share the final results. Findings will also be disseminated to various stakeholders including policy makers at a policy symposium held before the end of the study. The final trial report will be written by the researchers of ARTEMIS guided by the PI and CoIs. Professional writers will not be hired to write the trial report.

## Discussion

The ARTEMIS involves testing an anti-stigma campaign and an EDSS platform that will allow for identification, diagnosis, and treatment of depression, other significant emotional or medically unexplained complaints and risk of suicide/self-harm among adolescents living in urban slums. The key elements that ARTEMIS will focus on are increasing awareness among adolescents and the slum community on these conditions as well as strengthening the skills of existing primary healthcare workers and promote task sharing It is underpinned by the World Health Organizations’ (WHO) Mental Health Gap Action Programme (mhGAP) and builds on learnings from previous formative work. It includes a robust outcome evaluation design coupled with a process evaluation to understand how contextual factors drive adoption.

### Strengths and limitations

The programme strengths are that the intervention aligns with recommendations from the WHO’s Mental Health Action Plan 2013-20 and India’s National Mental Health Policy to develop innovative strategies that strengthen existing systems. It builds upon other research initiatives in LMICs including India, and perhaps is a one of its kind research, among adolescents in urban slums. The study will contribute to deepening the understanding of the challenges of implementing complex interventions in ‘real world’ settings, which are influenced by ground realities experienced by healthcare providers, and the adolescents themselves. The evidence generated will have the potential to inform decision-making for system planners on a scalable solution to increasing access to high-quality primary healthcare for adolescents at high risk of depression and suicide/self-harm. The main study limitation is that although the study will be conducted in two different regions (South and North) of India, the outcomes may only be generalisable to similar cultural contexts and settings.

### Significance

ARTEMIS is unique as the authors have not been able to identify any datasets for adolescents with mental illness in urban slum communities in India. This trial has potential to increase workforce capacity through supporting UPHC doctors and NPHWs, in the identification and management of adolescents who are at high risk for depression and suicide/self-harm. ARTEMIS also aims to destigmatise mental disorders and promote help-seeking behaviour and treatment adherence among adolescents. This strategy has great potential to improve mental health outcomes for adolescents living in urban slums and who are unable to access high-quality mental health care.

## Supplementary Information

**Additional file 1: ** Participant Information Sheet for Adults (parents/guardians/ASHAs/Doctors/Other stakeholders) /Adolescents in the community (Baseline/Intervention/ Post trial).

## Data Availability

Not applicable. Sponsor The study is being sponsored by The George Institute for Global Health, India whose registered office is located in Plot No. 58 & 59, Ground Floor, Saranya Building Nagarjuna Circle, Punjagutta, Hyderabad - 500 082. The sponsor played no part in study design; collection, management, analysis, and interpretation of data; writing of the report; and the decision to submit the report for publication

## Abbreviations

UPHC: Urban primary health centre
NPHW: Non-physician health workers
ASHAs: Accredited social health activists
mHealth: Mobile health
EDSS: Electronic decision support system
IEC: Information, education and communication
AEAG: Adolescent Expert Advisory Group
ANMs: Auxiliary Nurse Midwives
PHQ: Patient health questionnaire
mhGAP-IG: Mental health gap action programme- intervention guide
cRCT: Cluster randomised controlled trial
WHO: World Health Organization
KAB: Knowledge attitude and behaviour
BACE-TS: Barriers to access to care evaluation- treatment stigma
ICMR: Indian Council of Medical Research
HMSC: Health Ministry Screening Committee
LMIC: Low- and middle-income countries.
