# Supplementary material for: An intervention to reduce stigma and improve management of depression, risk of suicide/self-harm and other significant emotional or medically unexplained complaints among adolescents living in urban slums: protocol for the ARTEMIS project
Source: Trials. 2022 Jul 29;23:612. doi: 10.1186/s13063-022-06539-8 (PMC9336093; doi:10.1186/s13063-022-06539-8)
Supplement: Supplementary file 1 — Additional file 1: Participant Information Sheet for Adults (parents/guardians/ASHAs/Doctors/Other stakeholders) /Adolescents in the community (Baseline/Intervention/ Post trial). [file 13063_2022_6539_MOESM1_ESM.docx]

Study Title: Adolescents’ Resilience and Treatment Needs for Mental Health in Indian Slums (ARTEMIS)

Participant Information Sheet for Adults (parents/guardians/ASHAs/Doctors/Other stakeholders) /Adolescents in the community (Baseline/Intervention/ Post trial)

**Introduction**

**You are invited to take part in a study entitled “Adolescents’ Resilience and Treatment Needs for Mental Health in Indian Slums (ARTEMIS).**

This Participant Information Sheet has all the detailed information about the study. This document explains all the procedures involved in this study before you decide whether to take part in it. It is entirely your choice to participate in this study and if you decide to take part, you can change your mind and withdraw from the study at any point in time.

Feel free to ask any questions about any information in the document. If you need additional information, please do not hesitate to ask. You may also wish to discuss the study with a relative or friend or your local health worker before you decide to participate. If you wish to participate in this study you will need to sign the Consent Form and by signing this form, you indicate that you understand the information and that you agree to participate in the study.

**Purpose and Background**

The George Institute for Global Health which is the organisation that is conducting this study aims to explore associated risk and resilience factors for Common Mental Disorders like depression, anxiety, suicide risk and substance abuse among adolescents (10-19 years) living in urban slums of selected sites. Develop a community-based intervention strategy to reduce stigma and improve community behaviours toward adolescents with CMDs; and improve the treatment of adolescents at high risk of CMDs- stress, depression, suicide risk. A mobile based app will collect data about mental disorders and other associated factors affecting individuals and will help in identifying and treating those with a CMD. ASHAs and primary care doctors will provide the clinical care and independent researchers will help monitor and gather research data, periodically at baseline, 3 month, 6 month, 12 month periods. Stigma related to mental health will be discussed too. This study would be evaluated to assess if it is effective and beneficial to the community. The study objectives are based on the idea that a community-based anti-stigma campaign will lead to significant improvements in community behaviours toward adolescents with common mental disorders; and a mobile device-based decision support system will improve the treatment of adolescents at high risk of CMDs and lead to higher remission rates from depression and reduced suicide risk.

The study will be conducted in 60 wards/blocks in urban slums of Andhra Pradesh and Delhi in total, including this ward/block. The entire duration of the study is 3 years, but your direct involvement will be for 24 months during which there will be an awareness increasing campaign for common mental disorders, a population-based survey followed by intervention, and a post intervention follow-up.

**Procedures:**

We are inviting you to participate in this study because you have been screened as being at high risk for depression or are at high risk of suicide-risk or have been selected because you are a resident of this slum, in which the ARTEMIS study is being implemented

The study involves the following:

i)Initially a screening of adolescents for common mental disorders by our field staff who will ask adolescents a few questions. Based on their responses, the adolescents will be getting a score. If an adolescent gets a score of ≥10 on the patient health questionnaire (PHQ 9) or has a score of ≥ 2 on the suicide question they will be in the high risk group, which means that they will need some medical help.

ii) This will be followed by collecting information on other parameters by our field staff.

iii) Then, high-risk adolescents will be asked to see a doctor at their nearest UPHC for getting treatment which could be either counselling or medications or combination of both. This phase is the intervention phase and the status of the adolescents’ mental health condition will be monitored on a regular basis by the ASHA in your locality.

iv) After this phase adolescents and you as a parent/ASHA/doctor/other important stakeholder will be asked about your views/experiences about the overall conduct of the intervention. In this phase you will be participating in group discussions/in person interviews which will be conducted by our research staff

v) Some adolescents who are not at high risk will also be included and will be provided knowledge about mental health issues and stigma related to mental health.

An interview will require about half an hour to 45 minutes of your time. A focus group discussion will take about one hour. We would also like to inform you that interviews and focused group discussions will be audio recorded and transcribed, subject to you giving your consent to such recordings.

You are requested to provide your consent/assent if you agree to participate in the above-mentioned activities.

**Risks or discomforts on participation**

There is minimal risk to participants during participation in this study. However, it is possible that while responding to some questions you might experience distress; in such cases please let us know and we will refer you to a counsellor or other mental health professional based on need. We will provide you with contact details, if needed.

**Possible benefits on participation**

If you are at high risk of CMDs, the study will provide you with the opportunity to seek appropriate care through the primary health care system. For everyone, irrespective of their CMD risk the study will provide you with an understanding about the relationship between CMDs and associated risk factors among adolescents and help in reducing stigma in the community towards adolescents with CMDs and improve the treatment seeking behaviour for CMDs among adolescents.

**Privacy, Confidentiality and Disclosure of Information**

All the information collected from you for the study will be kept strictly confidential. Your information will be identified only by your unique study subject number, date of birth and initials. Only the researchers from the George Institute for Global Health and research ethics committees may have direct access to it. No report of the data or any publication of the findings will include information that would allow you to be identified.

**Participation is Voluntary**

Participation in this research study is voluntary. If you do not wish to participate you are not obliged to. If you decide to take part and later change your mind, you are free to withdraw from the study at any stage with no adverse consequences for you. This will in no way affect your treatment or care provided by the health care system. Before you make your decision, a member of the study team will be available to answer any questions you have. Should you wish to withdraw, you can convey your decision to the field staff or contact given below. Any data collected from you prior to your withdrawal will be deleted from our database.

**What if something goes wrong?**

The George Institute for Global Health holds insurance policies which apply to this study. If you experience serious harm as a result of taking part, you may be able to claim compensation.

**Contact details**

When you have read this information, the interviewers will discuss it with you further and will answer any questions you may have. If you would like to know more about this study at any stage, please feel free to contact:

| **Contact person:** | **Principal Investigator:** |
| --- | --- |
| Dr Y.K.Sandhya,  Senior Research Fellow,  The George Institute for Global Health  308, Third Floor, Elegance Tower Plot No. 8, Jasola District Centre New Delhi 110025  Phone 011 4158 8091-93  Email: syatirajula@georgeinstitute.org.in  Mrs. Sudha Kallakuri,  Research Fellow,  The George Institute for Global Health  Plot No. 58 & 59, Ground Floor, Saranya Building, Nagarjuna Circle, Punjagutta, Hyderabad - 500 082  Phone: 040 4972 4444  Email skallakuri1@georgeinstitute.org.in | Dr Pallab Maulik,  Deputy Director and Director of Research, The George Institute for Global Health  Plot No. 58 & 59, Ground Floor, Saranya Building, Nagarjuna Circle, Punjagutta,  Hyderabad - 500 082  Phone: 040 4972 4444  Email: pmaulik@georgeinstitute.org.in |

**Statement of approval:**

This study has received ethical approval from The George Institute Ethics Committee (TGIEC), New Delhi. Any person with concerns or complaints about the conduct of the research study can contact

| **TGIEC Ethics Committee:** |
| --- |
| Member Secretary Institutional Ethics Committee,  The George Institute Ethics Committee 311-312,  Third Floor, Elegance Tower, Plot No 8,  Jasola District Centre,  New Delhi, 110025  Telephone: 011- 41588091  Email: [membersecretary@georgeinstitute.org.in](mailto:membersecretary@georgeinstitute.org.in) |
